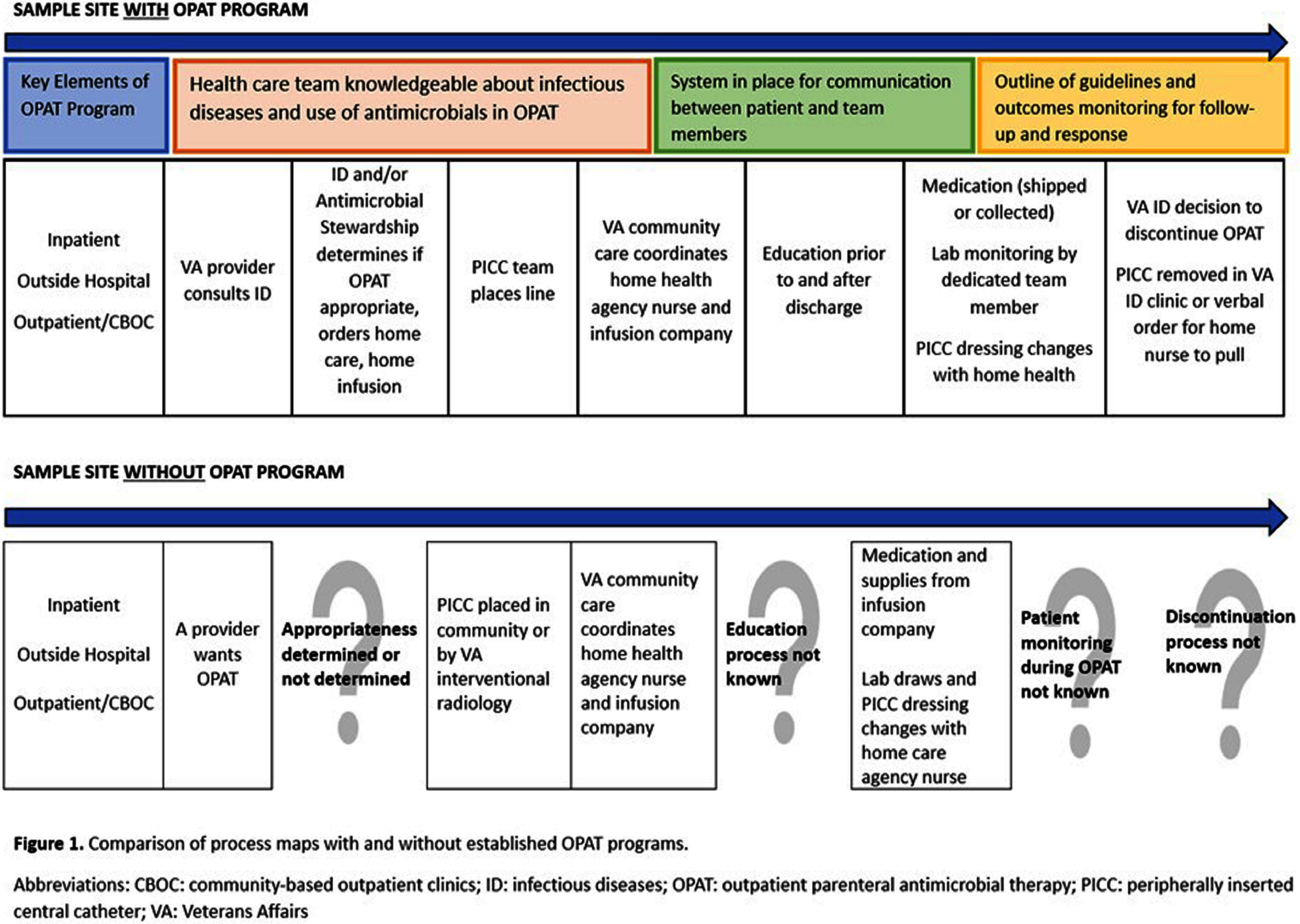# Provider Experience with Outpatient Parenteral Antimicrobial Therapy in Veterans Affairs Healthcare Systems: a qualitative study

**DOI:** 10.1017/ash.2025.262

**Published:** 2025-09-24

**Authors:** Elizabeth Scruggs-Wodkowski, Lauren Gauntlett, Amanda Blok, Danielle Helminski, Nicholas Henry, Sara Keller, Ronald Kendall, Manon Nitta, Allison Ranusch, Molly Harrod, Sarah Krein

**Affiliations:** 1 VA Ann Arbor Healthcare System; 2 University of Michigan Healthcare System; 3 Center for Clinical Management Research, VA Ann Arbor Healthcare System; 4 Johns Hopkins University School of Medicine

## Abstract

**Background:** Variability in outpatient parenteral antimicrobial therapy (OPAT) management and challenges to providing recommended OPAT care can compromise patient safety and care quality. Little is known about how OPAT is currently delivered by healthcare systems across the United States (US), including within the Veteran’s Health Administration (VHA). We sought to understand and compare OPAT delivery at selected Veterans Affairs medical centers. **Method:** Using a qualitative methodology, we conducted semi-structured interviews with key informants involved in OPAT delivery at 6 VHA medical centers with different complexity levels in the Midwestern US. Facility complexity is determined by patient volume and complexity level along with the amount of teaching and research conducted at the facility. Interviews occurred between February and December 2024 with healthcare personnel (n=30), including primary care and infectious diseases physicians, pharmacists, nursing staff, care coordinators, and vascular access providers. Data collection focused on better understanding OPAT processes within key domains of decision-making, patient education, care coordination, and post-discharge management. We used rapid analysis and a summary matrix to compare practices across sites within each domain. **Result:** Our findings highlight significant variability among VHA medical centers that provide OPAT to Veteran patients. Three of the 6 medical centers had dedicated OPAT programs as evidenced by a multidisciplinary team with clearly delineated roles and responsibilities, and processes that may help mitigate adverse outcomes and improve communication between providers at all OPAT care points. These processes map to the key elements outlined in the Infectious Diseases Society of America (IDSA) practice guidelines for OPAT programs, and include determination of appropriate therapy, patient education, lab monitoring, and discontinuation of treatment. (Figure 1) Conversely, at the three VHA sites without evidence of a multidisciplinary OPAT team or program, most participants described poor communication and coordination, lack of support, and uncertainty among providers about who is responsible for OPAT care. This confusion extends to follow-up and discontinuation of treatment. OPAT key elements were lacking or poorly defined. A process map helps visualize the contrasts in care between sites with and without defined OPAT programs. (Figure 1) **Conclusion:** Despite its centralized healthcare system, VHA medical centers demonstrate highly variable processes with respect to OPAT care. In the absence of a clear OPAT policy or program, uncertainty among providers about roles and responsibilities may be greater. The presence of a dedicated multidisciplinary OPAT team may help improve communication and care coordination, thereby minimizing quality and safety concerns.

**Figure:**